# Green Extraction of Natural Colorants from Food Residues: Colorimetric Characterization and Nanostructuring for Enhanced Stability

**DOI:** 10.3390/foods13060962

**Published:** 2024-03-21

**Authors:** Victoria Baggi Mendonça Lauria, Luciano Paulino Silva

**Affiliations:** 1 Embrapa Recursos Genéticos e Biotecnologia, Laboratório de Nanobiotecnologia (LNANO), Parque Estação Biológica, Final W5 Norte, Brasília 70770-917, DF, Brazil; victoriabaggi@gmail.com; 2 Programa de Pós-Graduação em Nanociência e Nanobiotecnologia, Universidade de Brasília, Campus Universitário Darcy Ribeiro, Brasília 70910-900, DF, Brazil

**Keywords:** food residues, green extraction, natural colorants, food colorants, RGB color model, nanotechnology

## Abstract

Food residues are a promising resource for obtaining natural pigments, which may replace artificial dyes in the industry. However, their use still presents challenges due to the lack of suitable sources and the low stability of these natural compounds when exposed to environmental variations. In this scenario, the present study aims to identify different food residues (such as peels, stalks, and leaves) as potential candidates for obtaining natural colorants through eco-friendly extractions, identify the colorimetric profile of natural pigments using the RGB color model, and develop alternatives using nanotechnology (e.g., liposomes, micelles, and polymeric nanoparticles) to increase their stability. The results showed that extractive solution and residue concentration influenced the RGB color profile of the pigments. Furthermore, the external leaves of *Brassica oleracea* L. *var. capitata f. rubra*, the peels of *Cucurbita maxima*, *Cucurbita maxima x Cucurbita moschata*, and *Beta vulgaris* L. proved to be excellent resources for obtaining natural pigments. Finally, the use of nanotechnology proved to be a viable alternative for increasing the stability of natural colorants over storage time.

## 1. Introduction

Colors are one of the most important sensory attributes in foods. They directly affect final product consumption since they serve as key criteria for judging the quality and even taste of foods by consumers [1]. Given the importance of colors in influencing eating behaviors, the industry uses artificial colorings (e.g., tartrazine, erythrosine, brilliant blue, and others) to intensify or preserve the initial colors of food during processing and storage stages [2,3]. This guarantee of product color quality control is carried out through color models, such as CIELab and RGB. The latter, for example, is capable of measuring the color of any object based on the sum of the variations in intensity between red, green, and blue [4]. The main advantages of using artificial dyes are their good stability to temperature, light, pH, and storage time, as well as their good coloring properties and low-cost production [2].

Regardless of the clear importance of artificial dyes, many have witnessed concerns about the potential adverse impacts of their production process on the environment and the possible risks to human health caused by their excessive intake [5,6]. On the other hand, there is a wide variety of natural pigments, such as anthocyanins, betanins, and carotenoids, which can replace artificial colorants. These biopigments are chemical compounds (metabolites) present in virtually all living organisms. Due to their rich structural diversity, they usually differ among themselves in terms of antioxidant, antineoplastic, anti-inflammatory, and antimicrobial properties [7,8,9].

In this scenario, by-products from industrial processes, specifically vegetable residues, such as peels, pomace, and seeds, among others, have been considered promising sources for developing natural food colorants due to their low cost and abundance in industrial, commercial, and household sectors, as well as the presence of health-beneficial phytopigments [10]. Furthermore, the reuse of food residues minimizes environmental pollution since their decomposition results in gas emissions that contribute to climate change [11].

Despite the environmental benefits associated with the use of food residues, obtaining these natural colorants generally requires organic solvents. Due to this fact, there is an increasing interest in technology development aimed at using alternative and nontoxic (green) solvents due to global environmental and climate imbalances [12]. That is why several studies have aimed at finding suitable sources for obtaining natural colorants from the use of sustainable technological innovations in correspondence with green chemistry principles [13,14].

Another challenge that needs to be overcome is the instability of natural compounds when subjected to environmental variations. In this sense, the use of nanotechnological strategies to minimize this degradation process appears as an emerging alternative, given that studies in the literature have already demonstrated the efficiency of nanosystems in protecting biopigments [15]. Nanotechnology refers to a field of technology responsible for manipulating materials, devices, and systems on a nanometric scale (10^−9^ m) [16]. Currently, in the food industry, nanotechnology has been used in several ways, such as in the nanoencapsulation of functional substances, aiming to improve the nutritional quality of food, as well as in the application of nanoparticles in packaging to increase the shelf life of foods [17,18].

In this context, this study innovates by developing an eco-friendly method to extract natural pigments from food residues and produce lipid and polymeric nanosystems to enhance the stability of these compounds. This pioneering approach opens new avenues for the food, pharmaceutical, and cosmetic industries, enabling the development of sustainable products.

## 2. Materials and Methods

### 2.1. Materials

In total, eight food residues were chosen as raw materials for the extraction of natural dyes, such as (i) hybrid tetsukabuto pumpkin peels (*Cucurbita maxima x Cucurbita moschata*); (ii) moranga pumpkin (*Cucurbita maxima*) peels; (iii) sweet potato peels (*Ipomoea potatoes*); (iv) beetroot peels (*Beta vulgaris* L.); (v) carrot peels (*Daucus carota* subsp. *sativus*); (vi) chayote shells (*Sechium edule Sw*); (vii) cabbage stalks (*Brassica oleracea* L. *var. acephala*); and (viii) purple cabbage external leaves (*Brassica oleracea* L. *var. capitata f. rubra*). These food residues were obtained through a donation made by the SESI industrial kitchen in the Federal District (DF) and/or by the team members of the Laboratory of Nanobiotechnology (LNANO) at Embrapa Genetic Resources and Biotechnology. Sodium hypochlorite (NaClO) (Agistereli Ltda, Itapevi, SP, Brazil) and sodium bicarbonate (NaHCO_3_) (Kitano, São Bernardo do Campo, SP, Brazil) were purchased at a local supermarket. Ethanol (Itajá, Goianésia, GO, Brazil) and ultrapure water were used to prepare the extraction solutions. Nonionic surfactant Tween 80 (Sigma-Aldrich, St. Louis, MO, USA), soy lecithin (Saint Charbel, Viçosa, MG, Brazil), glacial acetic acid (Merck, Darmstadt, Germany), chitosan from shrimp shells, ≥75% deacetylated (Sigma-Aldrich, St. Louis, MO, USA), and sodium tripolyphosphate (Sigma-Aldrich, St. Louis, MO, USA) were used for the development of nanosystems containing natural pigments.

### 2.2. Sanitization Process

Firstly, all vegetables were subjected to pre-washing with running water, aiming to eliminate the surface dirt. Then, they went through a sanitization process with 2.5% NaClO, remaining submerged for 15 min. Subsequently, the residues were immersed in a NaHCO_3_ solution at a concentration of 10 mg/mL for 15 min. Finally, they were rinsed with distilled water for 2 min, dried with a paper towel, and stored at −20 °C in plastic packaging until the extraction assays. 

### 2.3. Extraction of Natural Pigments

#### 2.3.1. Solvent Screening

The extraction of natural pigments was carried out using ultrapure water and ethanol 25% and 96% as solvents. The food residues were individually weighed (0.5 g) and cut into small pieces to increase the contact surface and optimize the extraction process. The pigment extractions were conducted in a 1:10 solids-to-solvent ratio. The samples were sonicated for 30 min at 40 kHz in an ultrasound bath and then magnetic stirred for 30 min. Finally, the food residues were separated from the extraction solvent by centrifugation at 1500× *g* for 10 min. The supernatants (natural pigments) were collected and stored at 2–8 °C for colorimetric evaluation. From the RGB analyses, the best solvents and food residues were chosen. From there, extractions varying the residue mass concentrations were carried out.

#### 2.3.2. Variation of Residue Mass Concentrations

The extraction of natural pigments was performed in triplicate using five different mass concentrations of food residues (3.3%, 5%, 10%, 20%, and 30%). The extractions were carried out in triplicate, following the same methodology described in Section 2.3.1. The evaluation of RGB color profiles was carried out the day after the extraction and storage process.

### 2.4. Colorimetric Characterization

The colorimetric characterization of natural pigments was carried out in triplicate and determined by the RGB color space model. A volume of 200 μL of each pigment was added to a 96-well microplate. The microplate was placed on top of an acrylic box illuminated by a LightPad containing an LED light with a cool white color temperature of 6500 Kelvin (K). The images were acquired with an adapted iPod-based BiO Assay system using the Experimental Assistant app (n3D BioSciences Inc., Houston, TX, USA). The digital images were analyzed through the image processing software ImageJ (version 1.52a), which measured the light intensity in the RGB spectrums.

### 2.5. Color Profile Statistical Analysis

The mean and standard deviation data of R, G, and B color intensities were subjected to analysis of variance (ANOVA) at a significance level of 5% (*p* < 0.05) with the Paleontological Statistics software package—PAST (version 2.7c). The results are presented as the arithmetic mean ± standard deviation of the mean.

### 2.6. Development of Lipid and Polymeric Nanosystems

Using the best solvents and residue concentrations pre-established in previous trials, new extractions were conducted to obtain an adequate volume of natural pigment to produce micelles, liposomes, and polymeric chitosan nanoparticles. The nanostructured natural pigments were stored at 2–8 °C.

#### 2.6.1. Micelles

Micelles were produced from natural pigment obtained from pumpkin peels (*Cucurbita maxima*). A total volume of 10 mL of pigment was added to 0.15 μL of the surfactant Tween 80. The sample was stirred until complete dissolution. Then, an aliquot was submitted as a rotary evaporator with an immersion bath at 50 °C. After the solvent evaporated, the sample was hydrated with ultrapure water. An empty control, without the presence of natural pigment, was also produced under the same conditions.

#### 2.6.2. Liposomes

Liposomes were produced by the lipid film hydration method from natural pigment obtained from the peels of hybrid pumpkin (*Cucurbita maxima x Cucurbita moschata*) and red beet (*Beta vulgaris* L.). A total volume of 4 mL of pigments was added to 0.020 g of soy lecithin. The samples were stirred until complete dissolution. Then, an aliquot was submitted as a rotary evaporator with an immersion bath at 50 °C. After the solvent evaporation, the films were hydrated with ultrapure water. Empty controls, without the presence of natural pigments, were also produced under the same conditions.

#### 2.6.3. Chitosan Polymeric Nanoparticles

Polymeric chitosan nanoparticles were produced from natural pigment obtained from the external leaves of purple cabbage (*Brassica oleracea* L. *var. capitata f. rubra*). A total volume of 2 mL of pigments was submitted as a rotary evaporator in 4 cycles (2 cycles of 1 h at 40 °C, followed by 2 cycles of 1 h at 50 °C). After the solvent evaporation, 2.0 mL of water acidified (0.1 mol/L) with glacial acetic acid was added. Subsequently, the solution was added to 0.004 g of medium molecular mass chitosan, remaining under stirring for 1 h. Then, a 1 mg/mL sodium tripolyphosphate solution was dripped gradually into the acidified solution. The formulation remained under constant stirring for 5 min after the end of dripping. Finally, it was subjected to the breaking of its particles using an ultraturrax at 21,500 rpm. An empty control, without the presence of natural pigment, was also produced under the same conditions.

### 2.7. Characterization of Nanostructured Pigments

The hydrodynamic diameter (HD) and the polydispersity index (PdI) of the nanosystems and their respective empty controls were evaluated using the dynamic light scattering (DLS) technique. The Zeta potential (ZP) was determined from its electrophoretic mobilities. The samples were diluted in a ratio of 1:10 in ultrapure water. The analyses were carried out on ZetaSizer Nano ZS equipment (Malvern Panalytical, Worcestershire, UK) by DLS and ZP. DLS analyses were performed at an angle of 173° using a He-Ne laser (4 mW) at a wavelength of 633 nm. Three measurements were taken of each sample at 25 °C in automatic run mode. The results are presented as the arithmetic mean ± standard deviation of the mean.

### 2.8. Evaluation of Colorimetric Stability

Colorimetric stability tests were carried out with natural pigments and their nanosystems stored at 2–8 °C protected from light for 26 days. Stability assessment occurred through RGB analysis carried out weekly, in triplicate, according to the methodology described in Section 2.4. The results are presented as the arithmetic mean ± standard deviation of the mean.

## 3. Results

### 3.1. Influence of Solvent on Natural Pigment Extraction

**Figure 1:**
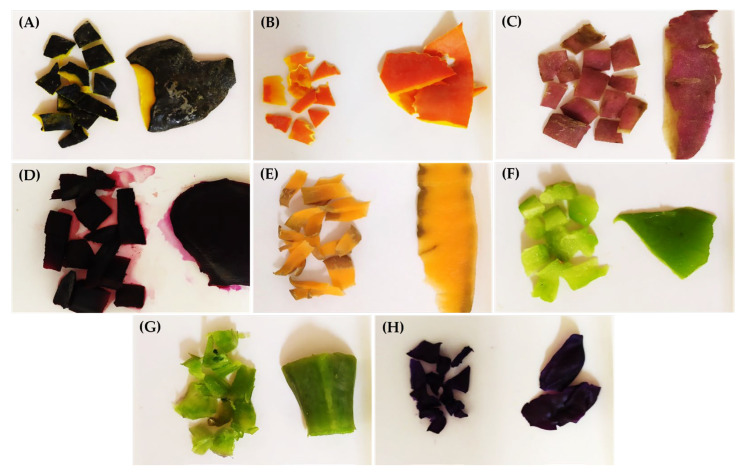
Food residues. (**A**) Tetsukabuto hybrid pumpkin peels; (**B**) moranga pumpkin peels; (**C**) sweet potato peels; (**D**) red beet peels; (**E**) carrot peels; (**F**) chayote shells; (**G**) cabbage stalks; (**H**) purple cabbage external leaves.

In total, eight food residues were chosen to extract natural pigments, as seen in Figure 1.

**Figure 2:**
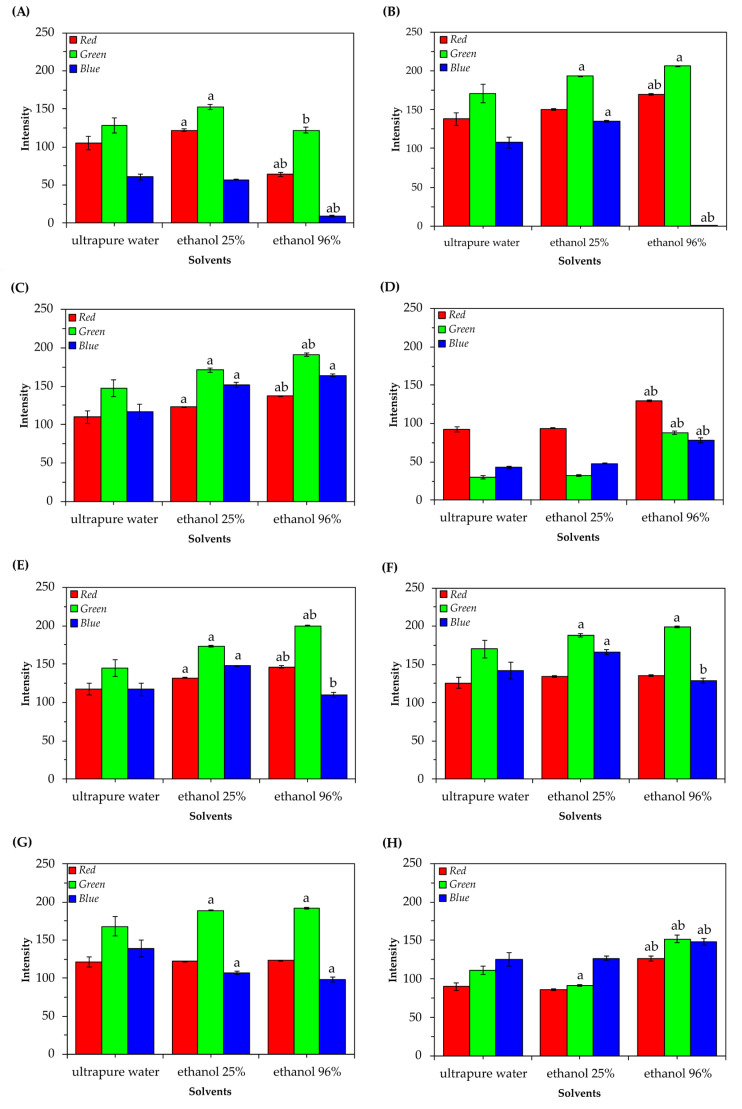
Average values (± standard deviation of the mean) of R’G’B’ of natural pigments obtained in different extractive solutions. (**A**) Tetsukabuto hybrid pumpkin peels; (**B**) moranga pumpkin peels; (**C**) sweet potato peels; (**D**) beetroot peels; (**E**) carrot peels; (**F**) chayote shells; (**G**) cabbage stalks; (**H**) purple cabbage external leaves. In each figure, columns with different letters indicate statistically significant differences at the 5% level by the Tukey Test, with “a” to water and “b” to ethanol 25% of the corresponding color.

To identify the optimal solvent for extracting the natural pigments, ultrapure water and ethanol 25% and 96% were used for extraction. Ethanol 96% showed to be the most suitable solvent for the extraction of natural pigments from residues of moranga pumpkin (*Cucurbita maxima*), carrot (*Daucus carota* subsp. *sativus*), hybrid tetsukabuto pumpkin (*Cucurbita maxima x Cucurbita moschata*), chayote (*Sechium edule Sw*), and cabbage (*Brassica oleracea* L. *var. acephala*) from RGB color profiles. It is possible to observe that the choice of extractive solution directly influenced the intensities of red, green, and blue. For example, the use of 25% or 96% ethanol solvents compared to water resulted in natural pigments with more pronounced green intensities, which are extracted from the peel of pumpkin, sweet potato, carrot, chayote, and cabbage stalks (Figure 2).

On the other hand, ethanol 25% proved to be a more promising alternative compared to ultrapure water and ethanol 96% for extracting the natural pigment obtained from the external leaves of purple cabbage (*Brassica oleracea* L. *var. capitata f. rubra*). A similar result can be found in a study carried out by Song et al. (2011) [19], where 25% ethanol was considered the best solvent for obtaining natural pigment from purple cabbage, compared to water, acetone, petroleum ether, and ethanol in higher concentrations.

Furthermore, it is possible to observe that both ultrapure water and ethanol at concentrations of 25% were good extractive solvents for obtaining natural pigments from red beet peels (*Beta vulgaris* L.) due to the similarities in the average RGB color profile (Figure 2). Similar results can be found in studies carried out by Zin et al. (2020) [20] and Kushwana et al. (2017) [21], who demonstrated the efficiency of ethanol and water solvents in extracting natural pigments obtained from the peels and bagasse of beetroot, respectively. Finally, none of the solvents proved to be efficient in extracting natural pigment from the peels of sweet potatoes (*Ipomoea potatoes*).

Although all residues present phytopigments of varied hues and tonal scales, the external leaves of purple cabbage and peels of moranga pumpkin, tetsukabuto hybrid pumpkin, and beetroot were considered sources of the greatest potential to continue this study owing to obtaining more intense dyes at the end of the extraction process. These more intense dyes have a wider range of applications and can be used to create new colors. Additionally, intense colors are more stable over time under favorable conditions. In addition, it was decided to use ultrapure water and ethanol 25% to extract the natural pigments present in beetroot peels and external leaves of purple cabbage, respectively. Finally, ethanol 96% was the solvent chosen to extract the natural pigments present in the peels of moranga pumpkin and tetsukabuto hybrid pumpkin, varying the residue mass concentrations.

### 3.2. Influence of Residue Mass Concentrations on Natural Pigment Extraction

**Figure 3:**
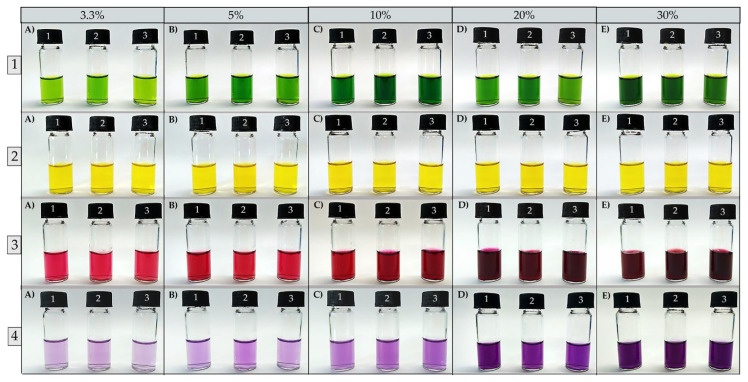
Natural pigments extracted from different concentrations of food residues (3.3%; 5%; 10%; 20; and 30%). (**1 A**–**E**) Pigments from tetsukabuto hybrid pumpkin peels; (**2 A**–**E**) pigments from moranga pumpkin peels; (**3 A**–**E**) pigments from beetroot peels; (**4 A**–**E**) pigments from purple cabbage external leaves.

Once the best food residues and their respective extractive solutions were identified, assays were carried out to verify the influence of the mass concentrations of residues in the extraction of natural pigments. It is possible to observe that the triplicates of the natural pigments presented different RGB color profiles, demonstrating that this variable affects the extraction process (Figure 3).

**Figure 4:**
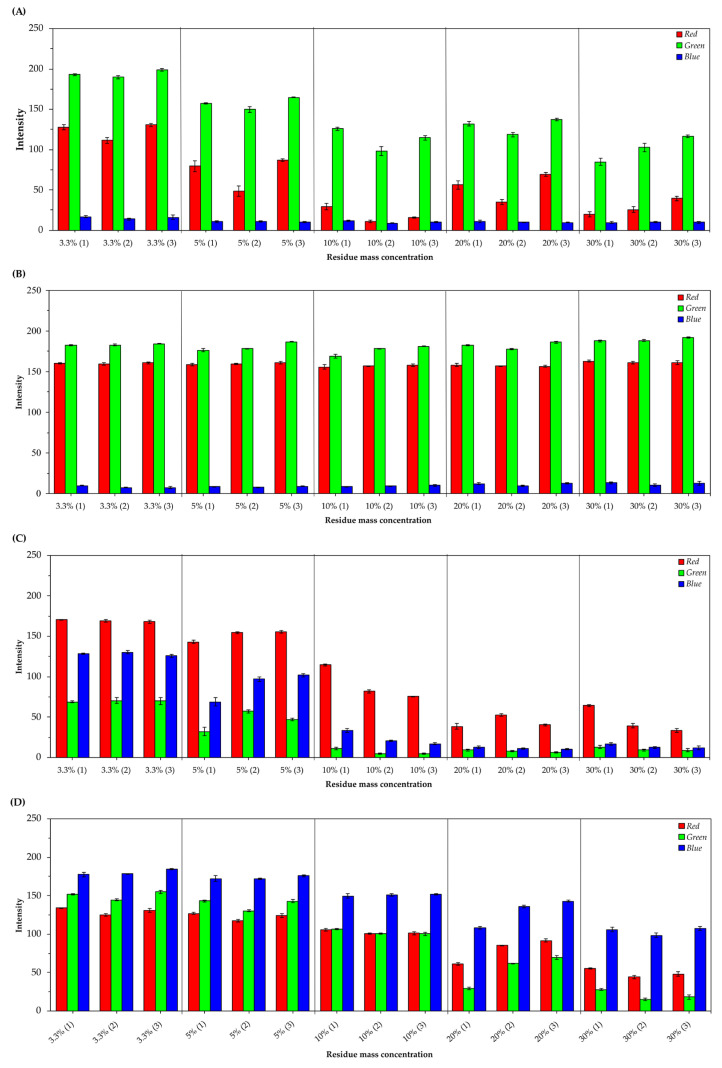
RGB average values (±standard deviation of the mean) of natural pigments triplicates extracted from different concentrations of waste (3.3%; 5%; 10%; 20%; and 30%). (**A**) Pigments obtained from tetsukabuto hybrid pumpkin peels; (**B**) pigments obtained from moranga pumpkin peels; (**C**) pigments obtained from beetroot peels; (**D**) pigments obtained from purple cabbage external leaves.

As seen in Figure 4, it is possible to notice that triplicates of natural pigments extracted from peels of tetsukabuto hybrid pumpkin (*Cucurbita maxima x Cucurbita moschata*) present high variations in red intensity for concentrations of 10%, 20%, and 30% of residue. The triplicates of natural pigments extracted from moranga pumpkin (*Cucurbita maxima*) peels showed small variations in the red and green intensities at all concentrations studied; only an average variation was observed in the blue intensity between the triplicates at the concentrations of 3.3%, 20%, and 30%. Furthermore, it is possible to observe that natural pigments extracted from beetroot peels (*Beta vulgaris* L.) showed greater heterogeneity in color patterns between concentrations of 5%, 10%, 20%, and 30%. Finally, small variations were observed in the intensities of red, green, and blue between triplicates at concentrations of 3.3%, 5%, and 10% for pigments extracted from the external leaves of purple cabbage (*Brassica oleracea* L. *var. capitata f. rubra*). Considering the 30% residue concentration yielded more intense colorants, it was selected for the nanostructuring process.

### 3.3. Hydrodynamic Diameter (HD), Polydispersity Index (PdI), and Zeta Potential (ZP) of Nanostructured Natural Pigments

The HD and PdI of the nanosystems were determined by DLS. This technique evaluates the size distribution (diameter) of particles and molecules in a liquid medium. In general, the movement of particles causes the incident light to be scattered with different intensities. It is from the analysis of these intensity fluctuations that the size of the particles is determined. The PdI offers information regarding the degree of sample dispersity, which can vary from 0 to 1. Values close to 1 indicate a highly polydisperse sample, while values close to 0 indicate homogeneity (monodisperse). The surface Zeta potential (ZP) of the particles was determined from their electrophoretic mobilities. The ZP values, represented in mV, indicate the colloidal stability of the suspension. ZP values greater than ±30 mV are indicative of colloidal dispersion results; ZP values lower than ±30 mV are indicative of colloidal instability [22]. A code designation was given to each nanostructure produced, as shown in Table 1.

The nanosystems containing natural pigments presented different sizes (HD), varying between 194.3 ± 19.0 nm and 645.2 ± 37.4 nm (Table 2). The average HDs exhibited by liposomes (194.3 ± 19.0 nm for LPCA and 273.0 ± 37.6 nm for LPCB) suggest structures consisting of only a phospholipid bilayer with a large internal aqueous compartment. The MPCA has an average HD of 645.2 ± 37.4 nm, providing the formation of structures with different dimensions already reported in the literature for the encapsulation of natural pigments [23].

Furthermore, size heterogeneity was observed for all nanosystems since the samples exhibited PdI ≥ 0.478 ± 0.043 (LPCB), as seen in Table 2. This high value may be caused by the presence of agglomerates or aggregates. An alternative to reducing this polydispersity is applying membrane separation methods, such as extrusion or high-pressure homogenization.

The nanosystems showed different colloidal stabilities. The NPQPFR showed incipient instability (24.4 ± 1.0 mV). In contrast, the LPCB, MPCA, and LPCA showed moderate (−35.9 ± 0.9 mV), good (−45.0 ± 0.7 mV), and excellent (−63.6 ± 0.1 mV) stability, respectively.

The MPCA and LPCA showed smaller average sizes and greater stability compared to their controls. When comparing the NPQPFR with its respective control, it is noted that the presence of the natural pigment obtained from the external leaves of purple cabbage enabled the formation of particles with smaller average sizes and homogeneous distribution. Finally, the presence of natural pigment extracted from beetroot peels resulted in the formation of liposomes with larger sizes and more monodispersity compared to their respective control. This increase in the nanosystem size can be explained by the fact that betalains (the main group of pigments present in beetroot peels) are relatively large molecules, which may explain the observed particulate growth.

### 3.4. Colorimetric Stability of Natural Pigments and Nanosystems

**Figure 5:**
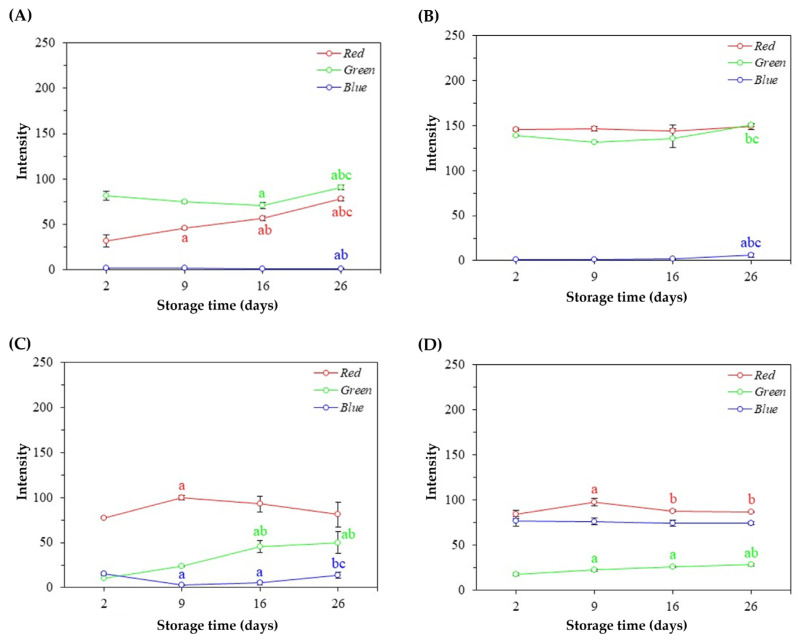
RGB average values (±standard deviation of the mean) of natural pigments during the storage time. (**A**) Pigments obtained from tetsukabuto hybrid pumpkin peels; (**B**) pigments obtained from moranga pumpkin peels; (**C**) pigments obtained from beetroot peels; (**D**) pigments obtained from purple cabbage external leaves. In each figure, different letters indicate statistically significant differences at the 5% level using the Tukey Test, with “a” in relation to 2 days, “b” in relation to 9 days, and “c” in relation to 16 days.

Significant variations (*p* < 0.05) were observed in the RGB profiles of natural pigments, as well as their respective nanosystems. However, a more pronounced RGB color variation was observed in the pigment from hybrid tetsukabuto pumpkin and beetroot peels (Figure 5A,C).

**Figure 6:**
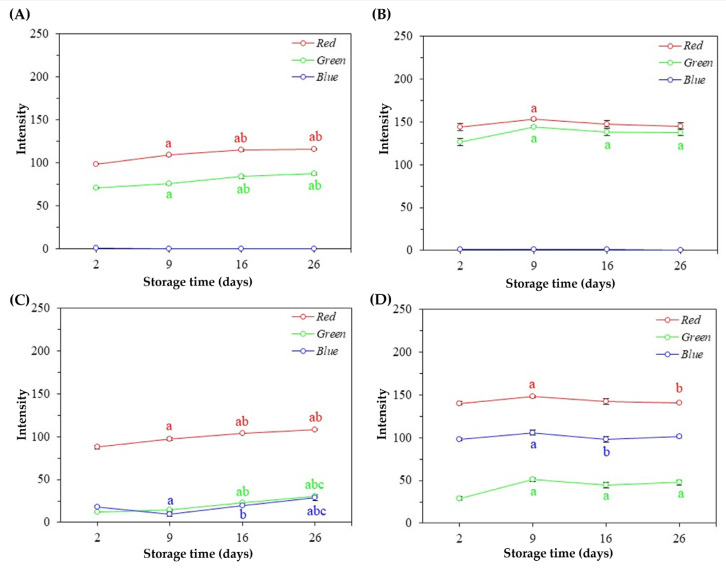
RGB average values (±standard deviation of the mean) of nanosystems during the storage time. (**A**) LPCA; (**B**) MPCA; (**C**) LPCB; (**D**) NPQPFR. In each figure, different letters indicate statistically significant differences at the 5% level using the Tukey Test, with “a” in relation to 2 days, “b” in relation to 9 days, and “c” in relation to 16 days.

The natural pigment extracted from tetsukabuto pumpkin peels showed significant changes (*p* < 0.05) in red intensity over time, indicating that this color is unstable even under favorable storage conditions (Figure 5A). Significant variations in the LPCA colorimetric profile were also observed, as shown in Figure 6A. However, from the 16th day of storage, variations in RGB values are not significant, signaling the color stability of this nanosystem.

Regarding the natural pigment obtained from moranga pumpkin peels, it is possible to observe a color difference only after the 26th day of storage (Figure 5B). About the micelles containing the pigment extracted from the same plant residue (MPCA), a significant difference was observed in the green color on the 9th, 16th, and 26th day of storage compared to the 2nd day, as well as in the red color on the 9th day compared to the 2nd day (Figure 6B). However, the RGB color profile of the nanostructured pigment did not show a significant difference between the 9th, 16th, and 26th days of storage, indicating colorimetric stability.

About the natural pigment extracted from beetroot peels, it is possible to notice significant variations in the green and blue intensities over time, signaling changes in the colorimetric profile, as shown in Figure 5C. In addition, the LPCB showed a significant variation in red, green, and blue intensities from the 9th day of storage, as seen in Figure 6C. Finally, no significant changes in blue intensity were observed in the natural pigment obtained from the purple cabbage’s external leaves (Figure 5D). On the other hand, significant differences in the intensities of red and green are observed in the first few days. Similar results were found in the nanostructured pigment (NPQPFR), as no significant changes in RGB intensities were observed between the 16th and 26th days of storage, signaling color stability (Figure 6D).

## 4. Discussion

The food industry commonly uses artificial colorings in its products, seeking to ensure more attractive sensory characteristics. However, the demand for healthier alternatives is growing in the market, and an alternative to meet this demand is the use of natural dyes. The exploitation of food waste as a source of natural dyes opens doors for innovation in the food industry, promoting sustainability and the valorization of resources that would previously be discarded. However, to ensure the viability of using food waste as a source of natural dyes, it is necessary to consider the application of appropriate and eco-friendly extractive methods. In this study, the eco-friendly extraction method of natural pigments using green solvents was explored.

The ultrasonic bath treatment followed by magnetic stirring has proved to be a simple, fast, and efficient green technological method for extracting natural pigments. The ultrasound technique has been widely applied mainly for the extraction of bioactive compounds due to its several advantages, such as its short time of operation, accuracy, and non-destructive method [24]. Higher extraction rates are commonly obtained using this technology due to the formation of acoustic cavitations in liquid media, thus generating greater penetration of the solvent into the plant matrix and release of intracellular content [25]. In the study conducted by Sharma and Bhat [26], ultrasound-assisted and microwave-assisted extractions were performed to obtain carotenoids. These extraction methods were compared with the conventional extraction method, and ultrasound-assisted extraction proved to be a more efficient technique due to its higher yields. The combination of the ultrasound technique with other extraction methods has recently become a focus in studies aimed at obtaining natural dyes due to the higher yields achieved [27].

The color variations observed during the extraction of natural pigments can be explained by the chemical structure of the potential classes of pigments extracted: (i) anthocyanins, present in the external leaves of purple cabbage and sweet potato peels; (ii) betalains, present in beetroot peels; (iii) carotenoids, present in pumpkin and carrot peels; and (iv) chlorophylls, present in cabbage stalks, chayote peels, and tetsukabuto hybrid pumpkin. The polarity of these natural compounds and the interactions between these pigments with carbohydrates, proteins, and other components in foods can influence the extraction process [28]. In addition, variations in the concentration of secondary metabolites affect the pigment hues. The content of secondary metabolites produced by a plant depends on several factors, such as temperature, humidity, light, altitude, macro- and micronutrients present in the soil, atmospheric composition, seasonality, plant genome, and mechanical stimuli, among others [29]. A promising alternative to avoid this variability in the color profile is the use of screening tests and phytochemical analyses to identify the chemical constituents’ values of interest. Furthermore, dilution and/or concentration processes can be adopted to achieve greater similarity in color between natural pigments.

Regarding the use of green solvents for the extraction of natural dyes, ethanol has proved to be an effective solvent, as it is possible to obtain attractive natural colorants from different food residues. In this way, it is possible to replace organic solvents with green alternatives to obtain dyes from natural sources, as can also be seen in the study by Li et al. (2013) [14].

The selection of the nanocarrier system for encapsulation was guided by the physicochemical properties of each dye. For instance, dyes extracted from moranga pumpkin peels are lipophilic and are thus frequently incorporated into micelles due to their affinity for hydrophobic environments. This affinity stems from the molecular structure of these compounds, which typically possess long non-polar hydrocarbon chains.

Micelles are aggregates of surfactant molecules that possess hydrophilic and hydrophobic moieties. The hydrophobic portions of the surfactant molecules cluster together at the core of the micelle, forming a hydrophobic environment [30]. The micelles produced in this study exhibit structures with different dimensions compared to those reported in the literature. This variation is influenced by some factors, including the chemical structure and concentration of the surfactant; the presence of counterion; and temperature variations during micelle formation [30].

The dye extracted from the external leaves of purple cabbage exhibits greater stability in an acidic environment. Therefore, chitosan polymeric nanoparticles emerge as a promising alternative to ensure this stability since chitosan, a natural biopolymer, is easily solubilized at acidic pH [31]. Among the methods for developing chitosan nanoparticles, ionic gelation stands out. This technique relies on the electrostatic interaction between the positively charged amino groups of chitosan and the negatively charged groups of a polyanionic substance, such as sodium tripolyphosphate (TPP), under acidic pH [31]. These nanoparticles can encapsulate and protect the dyes, reducing their degradation and increasing their resistance to photodegradation and other adverse conditions.

Dyes extracted from beetroot peels and tetsukabuto hybrid pumpkin peels are highly hydrophilic; therefore, a strategy for improving the stability of these compounds is their incorporation into liposomes. Liposomes are small, spherical vesicular structures formed by one or more phospholipid bilayers [32]. These nanosystems are composed of a non-polar tail consisting of fatty acid chains (hydrophobic region) and a polar head (hydrophilic region) formed by a phosphate group and a base (choline, glycerol, etc.). In the presence of water, these structures align, originating one or more concentric lipid bilayers, which are separated by an aqueous compartment [33]. Due to these characteristics, liposomes carry hydrophilic or lipophilic compounds.

Among the three nanotechnological strategies explored to enhance the stability of natural pigments, all nanosystems exhibited promising results. Compared to their unencapsulated counterparts, the pigments incorporated into these nanosystems displayed less color variation, indicating successful protection, particularly for pumpkin-derived pigments. The observed color changes in the LPCB and NPQPFR nanosystems can potentially be attributed to their high water content. Since these pigments are susceptible to nucleophilic attack by water molecules, the presence of water within the nanosystems could lead to alterations in color intensity [34]. Therefore, the application of water removal strategies, such as freeze-drying and spray-drying, could be valuable tools to further improve colorimetric stability [35].

## 5. Conclusions

Food residues (e.g., peels, stalks, and leaves) are promising sources to obtain natural pigments with varied color profiles. The extraction method is a simple and economical technique, proving to be a technological process with potential application on an industrial scale for obtaining natural pigments from different sources. In addition, the hydroethanolic extraction was very effective when performed with an ultrasound bath followed by magnetic stirring. This green solvent is an alternative to the use of organic solvents since there is a minimization of the negative impacts on both the environment and human health.

It is worth highlighting that studies of physicochemical characteristics and toxicity tests need to be explored to evaluate whether these natural colorants could be implemented in the food industrial sector at the stages of development, processing, and storage of foods, as well as in other industrial sectors, such as textiles, cosmetics, and inks, among others.

Furthermore, liposomes, micelles, and chitosan nanoparticles offer a compelling solution to overcome the challenge of rapid degradation observed with natural pigments during storage. Liposomes, formed by phospholipid bilayers, can encapsulate pigments, enhancing their stability and controlled release. Micelles also provide another encapsulation strategy, improving the water solubility and bioavailability of natural dyes. In addition, chitosan nanoparticles offer an attractive option to encapsulate stable dyes in an acidic environment. Their cationic nature allows for electrostatic interactions with the often negatively charged pigments, promoting efficient encapsulation and improved stability.

This research opens avenues for the further optimization of natural pigment extraction techniques, paving the way to explore new sources of natural dyes and aiming to meet the current market demand for more sustainable products.

## Figures and Tables

**Table 1:** Nanosystems containing natural pigments and their respective designations.

Food Residue	Nanosystem	Sample Designation
Tetsukabuto hybrid pumpkin peels	Micelles	MPCA
Moranga pumpkin peels	Liposomes	LPCA
Beetroot peels	Liposomes	LPCB
Purple cabbage external leaves	Chitosan polymeric nanoparticles	NPQPFR

**Table 2:** Average HD, PdI, and ZP (±standard deviation of the mean) of the nanosystems containing natural pigments extracted from different food residues and their respective empty controls.

Sample	HD Z-Average (nm)	PdI	ZP (mV)
Empty micelle control	604.1 ± 329.7	0.520 ± 0.039	−7.5 ± 1.8
MPCA	645.2 ± 37.4	0.802 ± 0.076	−45.0 ± 0.7
Empty liposome control	255.8 ± 44.4	0.524 ± 0.132	−46.3 ± 1.0
LPCA	194.3 ± 19.0	0.752 ± 0.009	−63.6 ± 0.1
LPCB	273.0 ± 37.6	0.478 ± 0.043	−35.9 ± 0.9
Empty control of polymeric chitosan nanoparticles	1022.7 ± 66.9	0.861 ± 0.046	43.3 ± 8.3
NPQPFR	228.8 ± 40.7	0.482 ± 0.060	24.4 ± 1.0

## Data Availability

The data presented in this study are available on request from the corresponding author due to intellectual property subjects.
